# Enhancing the Informed Consent Process in Pediatric Surgery Through an Educational Smartphone Application: Feasibility and Outcomes

**DOI:** 10.1155/ijta/5771756

**Published:** 2026-01-13

**Authors:** Cristian Bisanti, Marco Di Mitri, Francesco Di Carlo, Annalisa Di Carmine, Edoardo Collautti, Sara Maria Cravano, Sabrina Carta, Gaia Gazzo, Roberta Mauro, Greta Pichierri, Simone D’Antonio, Tommaso Gargano, Mario Lima

**Affiliations:** ^1^ Pediatric Surgery Department, AOU IRCCS Sant’Orsola-Alma Mater Studiorum, University of Bologna, Bologna, Italy, unibo.it

## Abstract

**Background:**

Informed consent (IC) is essential in medical decision‐making, ensuring that patients and their families fully understand the consequences of treatment options. In pediatric surgery, however, the process is often inadequate due to limited consultation time, high parental anxiety, and widespread misinformation. This study evaluates the effectiveness of the “ChPedBo” smartphone application in improving parental understanding and reducing preoperative anxiety.

**Methods:**

A prospective observational study was conducted at the Department of Pediatric Surgery, IRCCS Sant’Orsola‐Malpighi University Hospital of Bologna. A total of 150 questionnaires were distributed to parents of children scheduled for elective surgery; 100 completed questionnaires were included in the final analysis. Parents completed a structured 9‐item questionnaire assessing knowledge, anxiety, usefulness, and satisfaction before and after using the “ChPedBo” app. Statistical analysis included the Wilcoxon signed‐rank test, with a significance threshold set at *p* < 0.05.

**Results:**

Parental knowledge of their child’s condition significantly improved after using the app, with mean scores increasing from 2.39 to 3.14 (*p* = 3.88 × 10^−6^). Similarly, comprehension of the surgical procedure improved from 2.27 to 3.20 (*p* = 1.71 × 10^−6^). Preoperative anxiety levels decreased from 1.64 to 1.36 (*p* = 0.0073). The app was rated highly useful (mean score 2.64) and received a high overall satisfaction rating (mean score 2.70).

**Conclusions:**

The “ChPedBo” app significantly enhanced parental understanding and reduced preoperative anxiety, demonstrating its potential as a valuable digital tool in the IC process for pediatric surgery. Future studies should explore its application in emergency settings and its long‐term impact.

## 1. Introduction

Informed consent (IC) is a crucial process that ensures patients, and their families are aware of the consequences of their treatment decisions. In pediatric surgery, procedures can only be performed without consent in emergency situations when the decision‐maker—usually a parent or legal guardian—is unavailable [1].

Despite its critical importance, the process of obtaining IC is often inadequate, and parents’ comprehension of key elements of surgical consent is frequently poor. Providing complete and understandable information before an invasive procedure is an ethical requirement, and actively involving both the patient and parents in decision‐making is essential. Well‐informed patients and families tend to have higher satisfaction and are less likely to file legal claims [2].

The IC process primarily involves oral communication, during which the surgeon explains the indications, reasons for surgery, expected benefits, and step‐by‐step details of the procedure in a clear and simple manner. Alternative treatment options and the potential consequences of declining surgery should also be discussed. Additionally, parents must be informed about the postoperative period, including the need for medications (e.g., antibiotics and analgesics) and possible intraoperative and postoperative complications [3]. Discussions are typically guided by the four principles of biomedical ethics: autonomy, beneficence, nonmaleficence, and justice [4].

However, in daily clinical practice, only a few minutes are available for these discussions, and surgery‐related anxiety may further hinder parents’ ability to fully comprehend the information provided. Furthermore, the widespread availability of medical information on the internet has created a new challenge for healthcare professionals: misinformation and misinterpretation of online content. Parents frequently search the internet for additional details about their child’s condition and surgical options, but much of the available information is inaccurate, outdated, or not applicable to their specific case. Exposure to false or misleading medical content may lead to unrealistic expectations, increased anxiety, and distrust in medical recommendations, ultimately complicating the IC process [5]. The easy accessibility of online information has led to increased patient engagement, often resulting in detailed discussions that require the surgeon to carefully guide parents in interpreting medical data.

The widespread use of smartphones and immediate internet access has led to the development of numerous medical apps that assist physicians in their daily practice [6]. Considering the challenges in obtaining IC—including preoperative anxiety and the limited time available to convey complex information—we developed a smartphone application, available on Google Play and the Apple Store. This app provides structured, pathology‐specific information and is used for both routine and emergency surgery. The app is recommended after the IC discussion, not as a replacement for consent but as an educational support tool to reinforce comprehension and reduce anxiety. Parents remain free to modify or revoke their consent following additional information. Through the app, parents can also contact the medical team to ask additional questions before the procedure.

The aim of this study is to assess the role of the smartphone app in improving parental understanding of their child’s condition and surgical procedure, as well as its impact on anxiety levels and the amount of information effectively transmitted to parents.

## 2. Methods

### 2.1. Study Design

This was a prospective observational study aimed at evaluating the effectiveness of a smartphone application in improving parental understanding of their child’s condition and surgical procedure, as well as assessing its impact on parental anxiety, perceived usefulness, and overall satisfaction.

**Figure 1:**
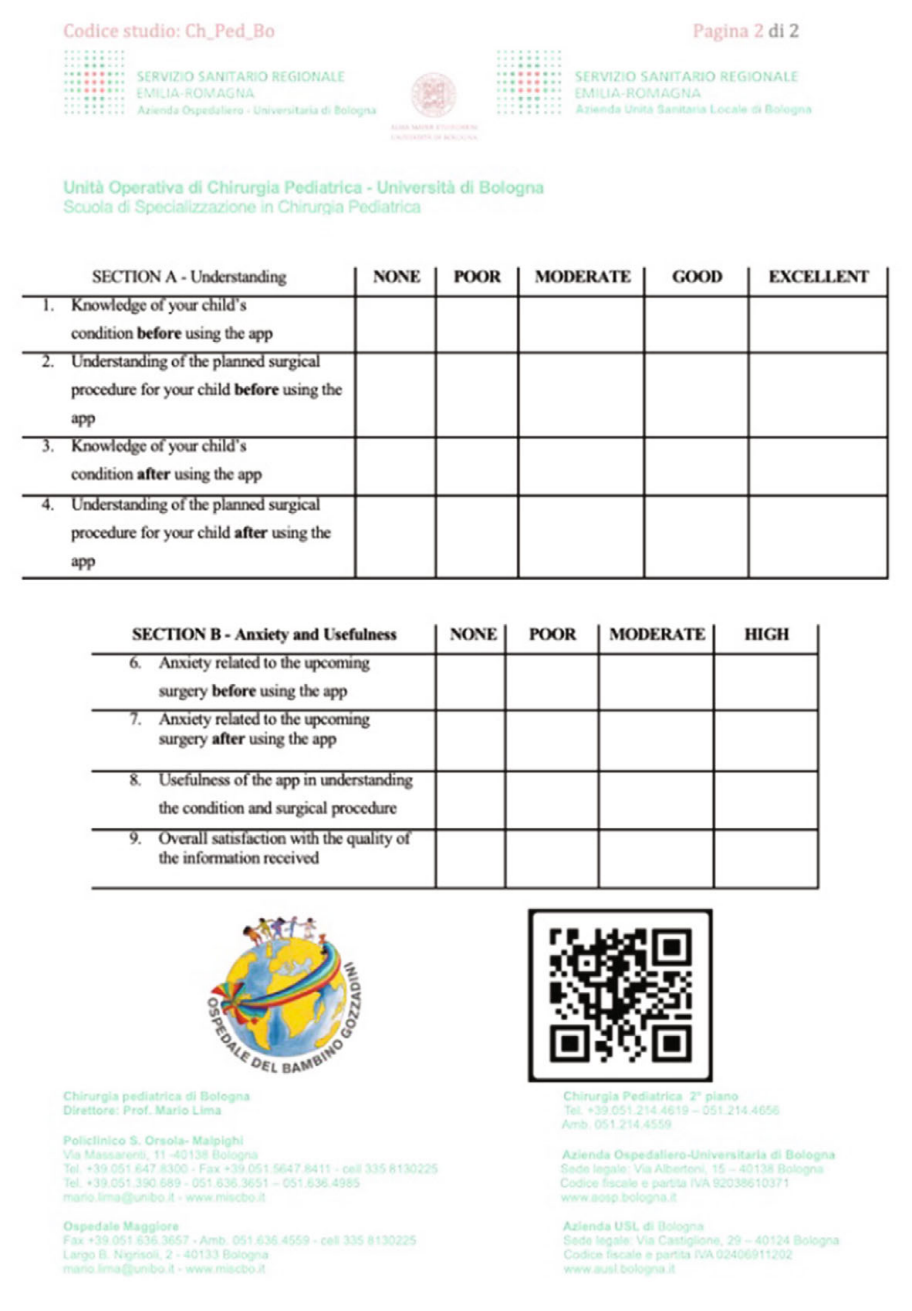
Questionnaire administered to the parents.

Parents of pediatric patients scheduled for elective surgery at the Department of Pediatric Surgery, IRCCS Sant’Orsola‐Malpighi University Hospital of Bologna, were invited to complete a questionnaire (Figure 1). This study was conducted in accordance with the ethical principles outlined in the Declaration of Helsinki and was approved by the institutional review board (IRB) of our hospital (approval code: “ChPedBo”). IC was obtained from all participants prior to their inclusion in the study. Only parents who expressed willingness to use the app were approached. Twenty parents declined and therefore did not receive the questionnaire.

Inclusion criteria consisted of the following:

1. Parents of children undergoing surgical procedures, and
2. Parents who expressed their intention to download the ChPedBo app.

**Figure 2:**
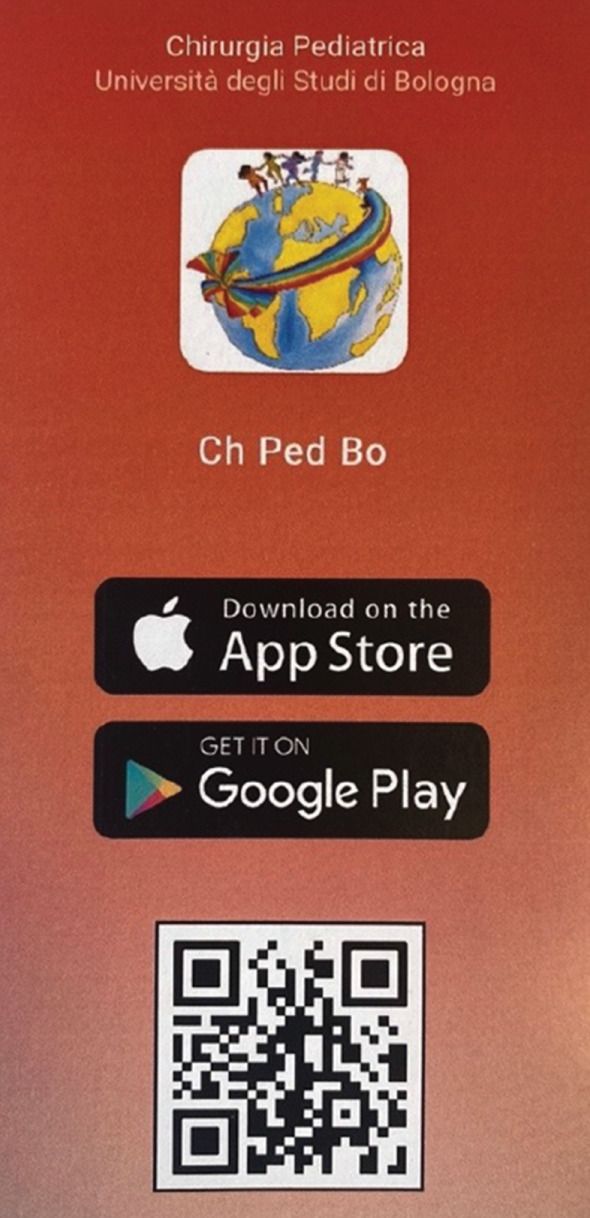
QR code to download directly the app.

At the end of the preoperative interview for elective surgery, all parents enrolled in the study were provided with a QR code, allowing them to download the app directly (Figure 2).

### 2.2. Questionnaire and Data Collection

Data were collected using a structured 9‐item questionnaire, divided into two sections:

• Section A—understanding: assessed parental knowledge and comprehension of the child’s pathology and surgical procedure before and after using the app. Responses were rated on a 5‐point scale (0–4).
• Section B—anxiety and usefulness: measured parental anxiety before and after app use, as well as the perceived usefulness and overall satisfaction with the app. Responses were rated on a 4‐point scale (0–3).

The questionnaire was administered immediately after the preoperative interview, with parents being asked to respond to questions regarding their baseline knowledge before surgery. After completing this section, they downloaded the app and returned the fully completed questionnaire on the day of surgery. The questionnaire used word‐based response labels (e.g., “poor,” “moderate,” “good,” and “excellent”). These categories were subsequently converted into numerical values (0–4 for Section A and 0–3 for Section B) exclusively for statistical analysis. The app does not substitute the formal IC discussion but complements it with additional structured information.

## 3. App

**Figure 3:**
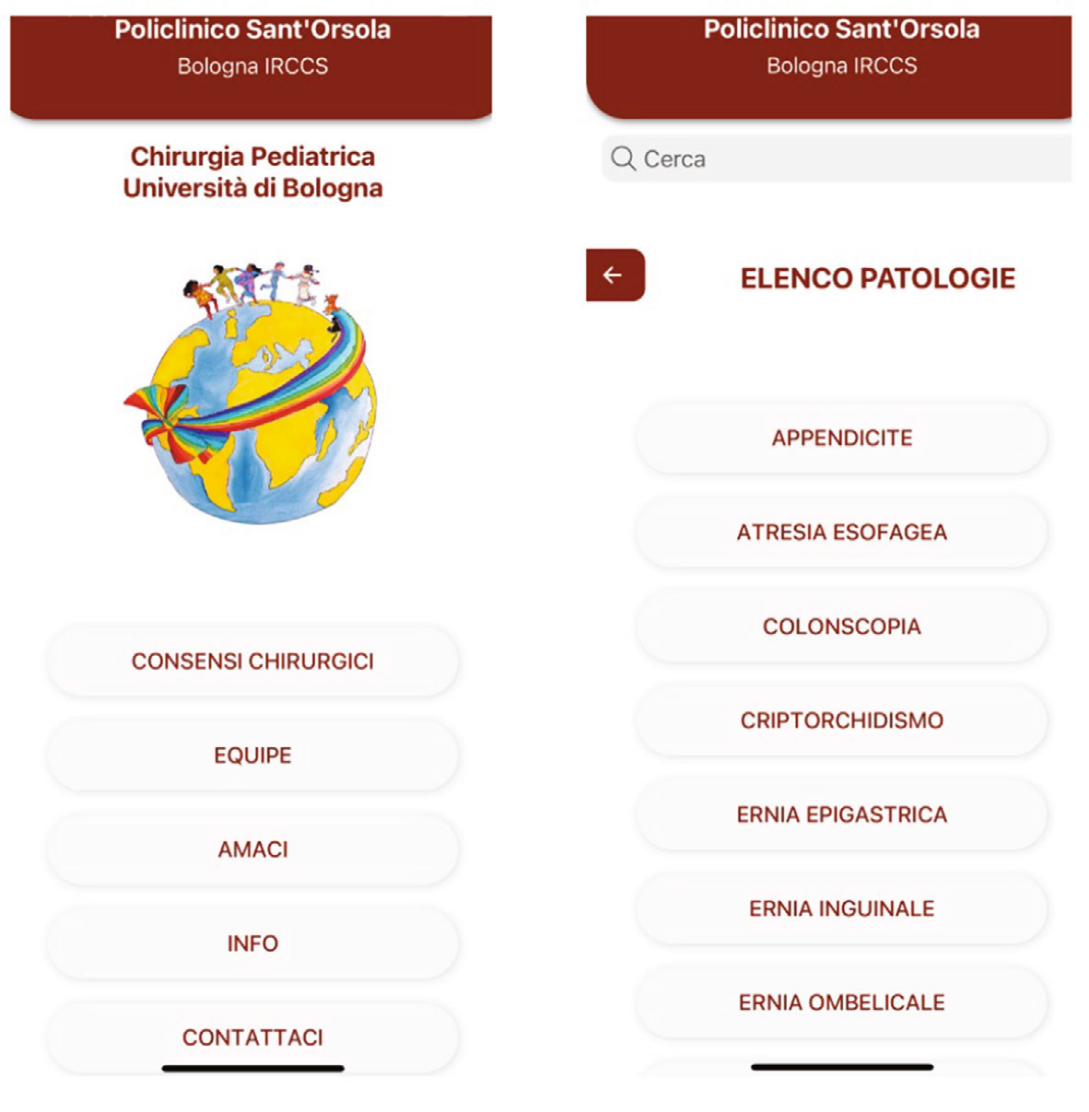
App interface.

We developed the “ChPedBo” app, available for free on Google Play and the Apple Store. “ChPedBo” is a hybrid mobile application built using the ionic capacitor framework, allowing for cross‐platform compatibility with a single code base (Figure 3).

The app is free to use and does not require user registration. It does not store or manage user data, ensuring privacy and security. At now, the app is available only in the EU app stores due to institutional restrictions.

Upon opening the app, the main menu displays the following options:

• “Informed Consent”
• “Equipe”
• “Legal Notes”
• “Contact Us”

By selecting “Informed Consent,” parents can access a list of pediatric surgical conditions. They can choose the relevant pathology for which surgery has been recommended and read all necessary information.

The “Equipe” section provides details about the surgical team, categorized by department: pediatric, infant, and newborn surgery.

Finally, through the “Contact Us” section, parents can submit questions before surgery by filling out a form. Once submitted, the inquiry is sent directly to our email inbox for review and response.

### 3.1. Statistical Analysis

Descriptive statistics were used to summarize participant responses. Continuous variables were reported as means and standard deviations. Normality of the data was assessed using the Shapiro–Wilk test. Given the nonnormal distribution, the Wilcoxon signed‐rank test was used to compare pre‐ and postapplication scores for knowledge, procedure understanding, and anxiety. A *p* value < 0.05 was considered statistically significant. Statistical analyses were conducted using the Microsoft Excel.

## 4. Results

The questionnaire was administered to parents immediately after the preoperative interview. A total of 150 questionnaires were distributed. Parents were asked to first respond based on their baseline knowledge and anxiety before downloading the app, then to use the “ChPedBo” app, and finally complete the postintervention section. Of the 150 distributed questionnaires, 100 fully completed forms were returned and included in the analysis. Fifty questionnaires were not returned. Common reasons included lack of time or loss of interest.

A total of 100 parents participated in the study, completing the questionnaire before and after using the “ChPedBo” app. Parents reported the procedure‐specific explanations as the most useful feature (82%), followed by information about the surgical team (65%) and the “Contact Us” function (48%).

### 4.1. Parental Understanding

Parental knowledge of their child’s condition significantly improved after using the app, with scores increasing from a mean of 2.39–3.14 (+31%) (*p* = 3.88 × 10^−6^). Similarly, comprehension of the surgical procedure improved from 2.27 to 3.20 (+41%) (*p* = 1.71 × 10^−6^). These results indicate that the app was effective in enhancing parental understanding.

### 4.2. Parental Anxiety

Preoperative anxiety levels showed a statistically significant decrease (−17%) after using the app, with scores dropping from 1.64 to 1.36 (*p* = 0.0073), suggesting that the app contributed to a reduction in parental anxiety.

### 4.3. Perceived Usefulness and Satisfaction

The app was rated highly useful (+31%), with a mean score of 2.64 (out of 3) and a median score of 3, indicating that most parents found it beneficial in improving their understanding of their child’s condition and surgery. General satisfaction with the app was also very high, with a mean score of 2.70 and a median of 3, demonstrating that nearly all parents were satisfied with the quality of information provided.

### 4.4. Overall Findings

The results indicate that the “ChPedBo” app significantly improved parental knowledge and understanding of their child’s condition and surgical procedure, while also contributing to a reduction in preoperative anxiety. Additionally, high levels of usefulness and satisfaction were reported, supporting the app’s role as a valuable tool in the IC process.

## 5. Discussion

IC is the foundation of medical decision‐making, and its absence constitutes an illegal act, carrying potential civil and criminal liability. Our findings show that a structured educational app can significantly improve parental understanding and reduce preoperative anxiety. This supports the use of digital educational tools as adjuncts to the IC discussion [7]. The magnitude of improvement and high satisfaction rates suggest that smartphone‐based structured information can compensate for the time constraints of standard consultations.

It is important to clarify that the “ChPedBo” application is not a digital IC platform. The app does not collect signatures, record decisions, or formalize legal consent. Instead, it functions purely as an educational adjunct, providing structured, pathology‐specific information to reinforce what parents have already discussed with the surgical team during the consent conversation. For this reason, the app is provided after the consent discussion, as an additional tool to consolidate understanding rather than as part of the legal consent workflow. This distinction differentiates our app from true digital consent systems which digitize the legal and procedural aspects of IC through structured pathways, multimedia modules, comprehension checks, and documented electronic consent. These platforms represent a fundamentally different technological domain from our tool, which focuses solely on enhancing comprehension through available educational content.

Recent literature, such as the paper by St John et al., has emphasized global considerations for digital IC and shared decision‐making in the digital era, highlighting both opportunities and challenges associated with fully digitized consent workflows [8]. In pediatric patients, consent for medical treatment is typically granted by parents or legal guardians. However, studies have shown that the comprehension of surgical procedures among parents is often suboptimal. Godzinsky et al. reported that 33% of parents desired more information regarding the surgical procedure, while 25% wanted more details on risks, benefits, and preoperative symptoms [8]. Conversely, Pfeil suggested that excessive information might contribute to increased parental anxiety [9].

IC has evolved from a paternalistic model to a patient‐centered approach, which supports the right of patients (or their guardians) to make autonomous healthcare decisions [10]. Parents have the right to the following:

• Refuse the proposed medical treatment or specific components of the treatment plan.
• Revoke previously given consent, even if doing so results in treatment discontinuation.

The IC process consists of four critical elements:

1. Provision of adequate information regarding the condition and proposed treatment.
2. Assessment of patient competence (or guardian competence) to make an informed decision.
3. Ensuring comprehension of the information provided.
4. Guaranteeing voluntary decision‐making, free from coercion.

Prior research has highlighted the challenges associated with IC in surgical settings. When a single surgeon explains a procedure related to a specific pathology, parental memory retention and comprehension of surgical risks tend to be uniformly poor [11]. Additionally, consent forms are often poorly structured and difficult to read, further complicating the process [12, 13]. In a systematic review, Falagas et al. found that only 29%–36% of parents adequately understood the surgical risks despite receiving preoperative explanations, and general satisfaction with the amount of provided information varied widely (58% in surgical settings and 80% in clinical research settings) [14].

The difficulty of ensuring comprehension during the IC process has been further emphasized by Ochieng et al., who found that many patients undergo surgery without knowing their surgeon’s identity or the reason for the procedure. In their study of 371 surgical patients, although 80% reported receiving explanations about their surgery, only 56.1% had all their questions answered, 17% did not know the type of surgery they had undergone, and another 17% had not explicitly given consent [15].

Interventions to improve IC quality have been explored. Chotai et al. conducted a systematic review analyzing the content and delivery of IC discussions, parental satisfaction, and comprehension. Their findings suggested that additional educational tools—including brochures, webpages, and smartphone apps—could enhance the process. They also recommended designated time for parental questions and optimization of the IC setting [16]. The rapid advancement of technology has further revolutionized patient education. Nwomeh et al. demonstrated that using a personal computer during IC discussions significantly improved parental comprehension and satisfaction [17]. Some hospitals have replaced traditional consent forms with computer‐based interfaces, where patients provide recorded consent instead of signing a physical document. However, this method often lacks verification of patient understanding, posing a potential risk [18, 19].

Innovative digital consent platforms have been explored to improve patient comprehension and retention. Leclercq et al. reviewed a computer‐based IC program that allows patients to access online educational content from home or the hospital. The program first assesses patient competence and then provides multimedia educational content, including text, audio explanations, and flash videos about their specific surgery. The patient’s level of understanding is evaluated, and if comprehension is insufficient, key points are repeated. Only after successfully completing this step does the consent process continue, ensuring adequate knowledge before signing the form [20].

With the widespread adoption of smartphones, digital tools have become even more accessible. The release of the first‐generation iPhone in 2007 and the subsequent emergence of Android devices led to exponential growth in smartphone usage. Today, there are an estimated 4.3 billion smartphones globally and approximately 325,000 healthcare‐related mobile apps available. These technological advancements present an opportunity to enhance the IC process, providing parents with structured, accessible, and reliable medical information while mitigating the risk of misinformation from unverified online sources [21].

The improvements observed in both knowledge and procedural understanding indicate that the app effectively reinforces information typically provided during the preoperative consultation. The reduction in anxiety further suggests that structured, accessible explanations help parents feel more prepared and reassured. These findings highlight the educational value of the tool in supporting the IC process, especially in contexts where time constraints may limit detailed in‐person explanations. Our findings suggest that digital solutions such as smartphone apps may serve as valuable tools in modernizing and optimizing IC discussions in pediatric surgery [22].

### 5.1. Limitations and Future Research

This study has some limitations, including a small sample size and the lack of long‐term follow‐up to assess the sustained impact of the “ChPedBo” app on parental comprehension and anxiety.

Validated shared decision‐making measures such as CollaboRATE or SDM‐Q9 were not included; future studies should integrate these tools to improve comparability with the broader literature. Furthermore, nonresponders (*n* = 50) could not be characterized, introducing potential selection bias.

Additionally, the study focused on elective surgeries, and further research is needed to evaluate its effectiveness in emergency settings. Finally, the app’s content is center‐specific and produced by our clinical team, which may limit scalability and standardization across institutions. Future studies should involve larger, multicenter trials and explore the integration of interactive features, such as multimedia content and real‐time physician communication, to further enhance the IC process.

## 6. Conclusions

The “ChPedBo” app significantly improved parental understanding of their child’s condition and reduced preoperative anxiety, demonstrating its value in the IC process. High levels of satisfaction and perceived usefulness highlight its role as a valuable digital tool in pediatric surgery. Integrating smartphone‐based solutions can enhance patient education and decision‐making, mitigating the risks of misinformation.

## Conflicts of Interest

The authors declare no conflicts of interest.

## Funding

This research received no specific grant from any funding agency in the public, commercial, or not‐for‐profit sectors.

## Data Availability

The data that support the findings of this study are available on request from the corresponding author.

## References

[1] Shah P. , Thornton I. , Kopitnik N. L. , and Hipskind J. E. , Informed Consent, StatPearls, 2025, StatPearls Publishing, Accessed March 24, 2025. http://www.ncbi.nlm.nih.gov/books/NBK430827/. 28613577

[2] Olejarczyk J. P. and Young M. , Patient Rights and Ethics, StatPearls, 2025, StatPearls Publishing, Accessed March 24, 2025. http://www.ncbi.nlm.nih.gov/books/NBK538279/. 30855863

[3] Parente G. , Di Mitri M. , D′Antonio S. , Cravano S. , Thomas E. , Vastano M. , Lunca R. , Gargano T. , Libri M. , and Lima M. , Pelvic Health Assessment in Adult Females Following Pediatric Appendicitis: A Monocentric Retrospective Case-Control Study, Children. (2022) 9, no. 3, 10.3390/children9030346, 35327718. PMC8946899 35327718

[4] Anderson O. A. and Wearne I. M. J. , Informed Consent for Elective Surgery—What Is Best Practice?, Journal of the Royal Society of Medicine. (2007) 100, no. 2, 97–100, 10.1258/jrsm.100.2.97, 2-s2.0-33847740709, 17277283. 17277283 PMC1791005

[5] McDool E. , Powell P. , Roberts J. , and Taylor K. , The Internet and Children’s Psychological Wellbeing, Journal of Health Economics. (2020) 69, 102274, 10.1016/j.jhealeco.2019.102274. 31887480

[6] Di Mitri M. , Parente G. , Bisanti C. , Thomas E. , Cravano S. M. , Cordola C. , Vastano M. , Collautti E. , di Carmine A. , Maffi M. , D’Antonio S. , Libri M. , Gargano T. , and Lima M. , Ask Doctor Smartphone! An App to Help Physicians Manage Foreign Body Ingestions in Children, Diagnostics. (2023) 13, no. 20, 10.3390/diagnostics13203285, 37892106. PMC10606892 37892106

[7] Interventions to Improve Patient Comprehension in Informed Consent for Medical and Surgical Procedures: An Updated Systematic Review - PMC, Accessed March 24, 2025. https://pmc.ncbi.nlm.nih.gov/articles/PMC7079202/. 10.1177/0272989X19896348 PMC7079202 31948345

[8] St John E. R. , Moore C. J. S. , Pillarisetti R. R. , and Spatz E. S. , Global Considerations for Informed Consent With Shared Decision-Making in the Digital Age, BMJ Evidence-Based Medicine. (2024) 29, no. 5, 346–349, 10.1136/bmjebm-2023-112740, 38697783. 38697783

[9] Gorodzinsky A. Y. , Hong P. , and Chorney J. M. , Parental Knowledge in Pediatric Otolaryngology Surgical Consultations: A Qualitative Content Analysis, International Journal of Pediatric Otorhinolaryngology. (2015) 79, no. 7, 1135–1139, 10.1016/j.ijporl.2015.05.013, 2-s2.0-84930540676, 26027724. 26027724

[10] Pfeil M. , Parents’ Experience of Giving Consent for Their Child to Undergo Surgery, Journal of Child Health Care. (2011) 15, no. 4, 380–388, 10.1177/1367493511410756, 2-s2.0-84860607448, 21996679. 21996679

[11] Loeff D. S. and Shakhsheer B. A. , The Ethics of Informed Consent and Shared Decision-Making in Pediatric Surgery, Seminars in Pediatric Surgery. (2021) 30, no. 5, 151101, 10.1016/j.sempedsurg.2021.151101, 34635277. 34635277

[12] Li F. X. , Nah S. A. , and Low Y. , Informed Consent for Emergency Surgery — How Much Do Parents Truly Remember?, Journal of Pediatric Surgery. (2014) 49, no. 5, 795–797, 10.1016/j.jpedsurg.2014.02.075, 2-s2.0-84901003325, 24851773. 24851773

[13] The Readability of Currently Used Surgical/Procedure Consent Forms in the United States - PubMed, Accessed March 24, 2025. https://pubmed.ncbi.nlm.nih.gov/9591001/. 10.1067/msy.1998.87236 9591001

[14] Paasche-Orlow M. K. , Taylor H. A. , and Brancati F. L. , Readability Standards for Informed-Consent Forms as Compared With Actual Readability, New England Journal of Medicine. (2003) 348, no. 8, 721–726, 10.1056/NEJMsa021212, 2-s2.0-0037456358, 12594317. 12594317

[15] Falagas M. E. , Korbila I. P. , Giannopoulou K. P. , Kondilis B. K. , and Peppas G. , Informed Consent: How Much and What Do Patients Understand?, American Journal of Surgery. (2009) 198, no. 3, 420–435, 10.1016/j.amjsurg.2009.02.010, 2-s2.0-69249116175. 19716887

[16] Ochieng J. , Buwembo W. , Munabi I. , Ibingira C. , Kiryowa H. , Nzarubara G. , and Mwaka E. , Informed Consent in Clinical Practice: Patients’ Experiences and Perspectives Following Surgery, BMC Research Notes. (2015) 8, no. 1, 10.1186/s13104-015-1754-z, 2-s2.0-84949684034, 26653100. PMC4675036 26653100

[17] Stefani B. , Codrich D. , Caputo M. R. , Guida E. , Scarpa M. G. , Boscarelli A. , and Schleef J. , Surgical Informed Consent in Elective Pediatric Surgery: Medical Inadequacy or Parental Scotomization?, 2023, Bianca, 10.20944/preprints202302.0219.v1.

[18] Nwomeh B. C. , Hayes J. , Caniano D. A. , Upperman J. S. , and Kelleher K. J. , A Parental Educational Intervention to Facilitate Informed Consent for Emergency Operations in Children, Journal of Surgical Research. (2009) 152, no. 2, 258–263, 10.1016/j.jss.2008.01.008, 2-s2.0-61849106563, 18374948. 18374948

[19] Bollschweiler E. , Apitzsch J. , Obliers R. , Koerfer A. , Mönig S. P. , Metzger R. , and Hölscher A. H. , Improving Informed Consent of Surgical Patients Using a Multimedia-Based Program?: Results of a Prospective Randomized Multicenter Study of Patients Before Cholecystectomy, Annals of Surgery. (2008) 248, no. 2, 205–211, 10.1097/SLA.0b013e318180a3a7, 2-s2.0-49849101677, 18650629. 18650629

[20] Eggers C. , Obliers R. , Koerfer A. , Thomas W. , Koehle K. , Hoelscher A. H. , and Bollschweiler E. , A Multimedia Tool for the Informed Consent of Patients Prior to Gastric Banding, Obesity. (2007) 15, no. 11, 2866–2873, 10.1038/oby.2007.340, 2-s2.0-38049085801, 18070779. 18070779

[21] Leclercq W. K. G. , Keulers B. J. , Scheltinga M. R. M. , Spauwen P. H. M. , and van der Wilt G. J. , A Review of Surgical Informed Consent: Past, Present, and Future. A Quest to Help Patients Make Better Decisions, World Journal of Surgery. (2010) 34, no. 7, 1406–1415, 10.1007/s00268-010-0542-0, 2-s2.0-77955469587, 20372902. 20372902 PMC2895877

[22] Kernebeck S. , Busse T. S. , Böttcher M. D. , Weitz J. , Ehlers J. , and Bork U. , Impact of Mobile Health and Medical Applications on Clinical Practice in Gastroenterology, World Journal of Gastroenterology. (2020) 26, no. 29, 4182–4197, 10.3748/wjg.v26.i29.4182, 32848328. 32848328 PMC7422538

